# Critical care management of severe traumatic brain injury in adults

**DOI:** 10.1186/1757-7241-20-12

**Published:** 2012-02-03

**Authors:** Samir H Haddad, Yaseen M Arabi

**Affiliations:** 1 Surgical Intensive Care Unit, Intensive Care Department, King Abdulaziz Medical City, PO Box 22490, Riyadh 11426, K.S.A; 2 Intensive Care Department, College of Medicine, King Saud Bin Abdulaziz University for Health Sciences, King Abdulaziz Medical City, PO Box 22490, Riyadh 11426, K.S.A

**Keywords:** Traumatic brain injury, head injury, head trauma, critical care

## Abstract

Traumatic brain injury (TBI) is a major medical and socio-economic problem, and is the leading cause of death in children and young adults. The critical care management of severe TBI is largely derived from the "Guidelines for the Management of Severe Traumatic Brain Injury" that have been published by the Brain Trauma Foundation. The main objectives are prevention and treatment of intracranial hypertension and secondary brain insults, preservation of cerebral perfusion pressure (CPP), and optimization of cerebral oxygenation. In this review, the critical care management of severe TBI will be discussed with focus on monitoring, avoidance and minimization of secondary brain insults, and optimization of cerebral oxygenation and CPP.

## Introduction

Severe traumatic brain injury (TBI), defined as head trauma associated with a Glasgow Coma Scale (GCS) score of 3 to 8 [1], is a major and challenging problem in critical care medicine. Over the past twenty years, much has been learned with a remarkable progress in the critical care management of severe TBI. In 1996, the Brain Trauma Foundation (BTF) published the first guidelines on the management of severe TBI [2] that was accepted by the American Association of Neurological Surgeons and endorsed by the World Health Organization Committee in Neurotraumatology. The second revised edition was published in 2000 [3] with an update in 2003, and the 3^rd ^edition was published in 2007 [4]. Several studies have reported the impact of implementation of guidelines-based management protocols for severe TBI on patient's treatment and outcome [5,6]. These studies have clearly demonstrated that the implementation of protocols for the management of severe TBI, incorporating recommendations from the guidelines, is associated with substantially better outcomes such as mortality rate, functional outcome scores, length of hospital stay, and costs [7,8]. However, there is still considerable and wide institutional variation in the care of patients with severe TBI.

In general, TBI is divided into two discrete periods: primary and secondary brain injury. The primary brain injury is the physical damage to parenchyma (tissue, vessels) that occurs during traumatic event, resulting in shearing and compression of the surrounding brain tissue. The secondary brain injury is the result of a complex process, following and complicating the primary brain injury in the ensuing hours and days. Numerous secondary brain insults, both intracranial and extracranial or systemic, may complicate the primarily injured brain and result in secondary brain injury. Secondary, intracranial brain insults include cerebral edema, hematomas, hydrocephalus, intracranial hypertension, vasospasm, metabolic derangement, excitotoxicity, calcium ions toxicity, infection, and seizures [9,10]. Secondary, systemic brain insults are mainly ischemic in nature [9,11], such as:

- Hypotension (systolic blood pressure [SBP] < 90 mm Hg)

- Hypoxemia (PaO_2 _< 60 mm Hg; O_2 _Saturation < 90%)

- Hypocapnia (PaCO_2 _< 35 mm Hg)

- Hypercapnia (PaCO_2 _> 45 mm Hg)

- Hypertension (SBP > 160 mm Hg, or mean arterial pressure [MAP] > 110 mm Hg)

- Anemia (Hemoglobin [Hb] < 100 g/L, or hematocrit [Ht] < 0.30)

- Hyponatremia (serum sodium < 142 mEq/L)

- Hyperglycemia (blood sugar > 10 mmol/L)

- Hypoglycemia (blood sugar < 4.6 mmol/L)

- Hypo-osmolality (plasma osmolality [P Osm] < 290 mOsm/Kg H_2_O)

- Acid-base disorders (acidemia: pH < 7.35; alkalemia: pH > 7.45)

- Fever (temperature > 36.5°C)

- Hypothermia (temperature < 35.5°C)

Hence, it is now clear that only part of the damage to the brain during head trauma is from the primary brain injury, which is not amenable to alteration and cannot be reversed. However, secondary brain insults are often amenable to prevention or reversal.

**Figure 1:**
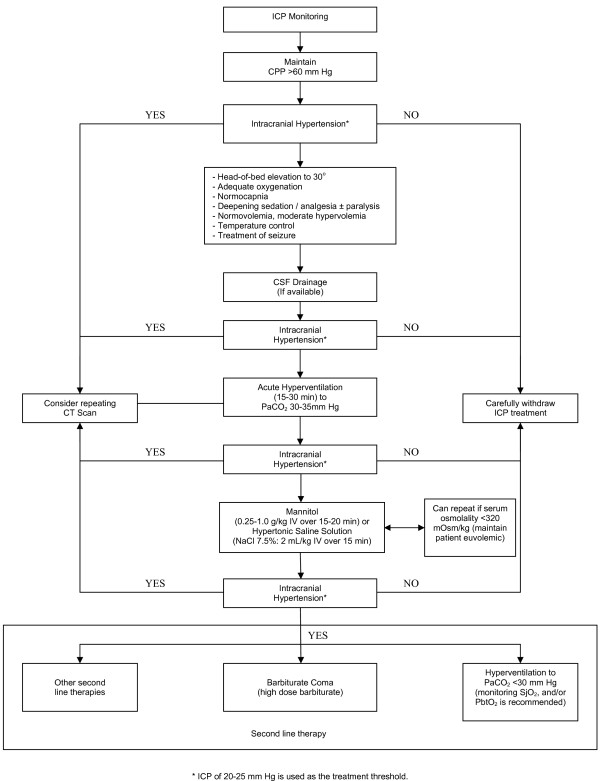


The intensive care management of patients with severe TBI is a dynamic process, starts in the pre-hospital period, at the scene of the accident. During the early stages of hospital care, the patients may be managed in a variety of locations including emergency department, the radiology department, and the operating room before they are admitted to the Intensive Care Unit (ICU). The continuum of acute care, during the "GOLDEN HOUR", from the time of injury through the start of definitive care, should be ensured and based on the guidelines and recommendations previously mentioned. This review outlines the fundamental principles of critical care management of patients with severe TBI during their stay in the ICU. See Figure 1

### Critical care management of severe TBI

Prior to arrival to the ICU, patients with severe TBI are usually received, resuscitated and stabilized in emergency department or operating room. Once the severely head-injured patient has been transferred to the ICU, the management consists of the provision of high quality general care and various strategies aimed at maintaining hemostasis with:

- Stabilization of the patient, if still unstable

- Prevention of intracranial hypertension

- Maintenance of an adequate and stable cerebral perfusion pressure (CPP)

- Avoidance of systemic, secondary brain insults (SBI)

- Optimization of cerebral hemodynamic and oxygenation

### Monitoring

Monitoring of patients with severe TBI is essential for the guidance and optimization of therapy. The rationale of monitoring is early detection and diagnosis of secondary brain insults, both systemic and intracranial. Therefore, monitoring of patients with severe TBI must comprise both general and specific neurologic monitoring.

### General monitoring

During neurointensive care of patients with severe TBI, general parameters that are regularly monitored include electrocardiography (ECG monitoring), arterial oxygen saturation (pulse oxymetry, SpO_2_), capnography (end-tidal CO_2_, PetCO_2_), arterial blood pressure (arterial catheter), central venous pressure (CVP), systemic temperature, urine output, arterial blood gases, and serum electrolytes and osmolality. Invasive or non-invasive cardiac output monitoring may be required in hemodynamically unstable patients who do not respond to fluid resuscitation and vasopressors.

### Neuromonitoring

#### Intracranial pressure monitoring

The BTF recommends that "intracranial pressure (ICP) should be monitored in all salvageable patients with a severe TBI and an abnormal computed tomography (CT) scan". Also, "ICP monitoring is indicated in patients with severe TBI with a normal CT scan if two or more of the following features are noted at admission: age over 40 years, unilateral or bilateral motor posturing, or systolic blood pressure (BP) < 90 mm Hg" [4]. Based on physiological principles, potential benefits of ICP monitoring include earlier detection of intracranial mass lesion, guidance of therapy and avoidance of indiscriminate use of therapies to control ICP, drainage of cerebrospinal fluid (CSF) with reduction of ICP and improvement of CPP, and determination of prognosis.

Currently, available methods for ICP monitoring include epidural, subdural, subarachnoid, parenchymal, and ventricular locations. Historically, ventricular ICP catheter has been used as the reference standard and the preferred technique when possible. It is the most accurate, low-cost, and reliable method of monitoring ICP [4]. It also allows for continuous measurement of ICP and for therapeutic CSF drainage in the event of intracranial hypertension to control raised ICP. Subarachnoid, subdural, and epidural monitors are less accurate. ICP monitor is usually placed via the right side, since in approximately 80% of the populations the right hemisphere is the non-dominant, unless contraindicated [12]. However, it might be placed on the side with maximal pathological features or swelling [13]. Routine ventricular catheter change or prophylactic antibiotic use for ventricular catheter placement is not recommended to reduce infection [4]. However, ICP monitoring devices are usually continued for ≤1 week; with daily examination of the CSF for glucose, protein, cell count, Gram stain, and culture and sensitivity. Treatment for intracranial hypertension should be started with ICP thresholds above 20 mm Hg. Additional to ICP values, clinical and brain CT findings should be used to determine the need for treatment [4].

Although there is no randomized, controlled trial (RCT) that has been performed demonstrating that ICP monitoring improves outcome or supporting its use as standard; ICP monitoring has become an integral part in the management of patients with severe TBI in most trauma centers. However, there is contradicting evidence about whether ICP monitoring improves outcome. Several studies have demonstrated that ICP monitoring reduced the overall mortality rate of severe TBI [14-21]. Other studies have not shown benefits from ICP monitoring [22-24]. Moreover, a few studies have demonstrated that ICP monitoring was associated with worsening of survival [25,26]. Potential complications of ICP monitoring include infection, hemorrhage, malfunction, obstruction, or malposition. Recently, we reported that in patients with severe TBI, ICP monitoring was not associated with reduced hospital mortality, however, with a significant increase in mechanical ventilation duration, need for tracheotomy, and ICU length of stay [27]. In the Cochrane database, a recent systematic review found no RCTs that can clarify the role of ICP monitoring in acute coma whether traumatic or non-traumatic [26]. Nevertheless, there is evidence, and most clinicians agree, to support the use of ICP monitoring in severe TBI patients at risk for intracranial hypertension. Absolute ICP values are independent predictors of neurologic outcomes; however, refractory ICP and response to treatment of raised ICP could be better predictors of neurological outcome than absolute ICP values [28]. Treggiari et al. conducted a systematic review to estimate the association between ICP values and patterns and short- and long-term vital and neurological outcome. Relative to normal ICP (< 20 mm Hg), raised ICP was associated with elevated odds ratio (OR) of death: 3.5 [95%CI: 1.7, 7.3] for ICP 20-40, and 6.9 [95% CI: 3.9, 12.4] for ICP > 40 mm Hg. Raised but reducible ICP was associated with a 3-4-fold increase in the ORs of death or poor neurological outcome. Refractory ICP pattern was associated with a dramatic increase in the relative risk of death (OR = 114.3 [95%CI: 40.5, 322.3]) [29].

#### Jugular bulb venous oxygen saturation

The jugular venous oxygen saturation (SjvO_2_) is an indicator of both cerebral oxygenation and cerebral metabolism, reflecting the ratio between cerebral blood flow (CBF) and cerebral metabolic rate of oxygen (CMRO_2_). A retrograde catheterization of the internal jugular vein (IJV) is used for SjvO_2 _monitoring. As the right IJV is usually dominant [30], it is commonly used for cannulation to reflect the global cerebral oxygenation [31]. Monitoring SjvO_2 _can be either continuous via a fiberoptic catheter or intermittent via repeated blood samples. In a prospective study of patients with severe acute brain trauma and intracranial hypertension, Cruz concluded that continuous monitoring of SjvO2 was associated with improved outcome [32]. The normal average of the SjvO_2_, in a normal awake subject, is 62% with a range of 55% to 71%. A sustained jugular venous desaturation of < 50% is the threshold of cerebral ischemia and for treatment [33]. SjvO_2 _monitoring can detect clinically occult episodes of cerebral ischemia, allowing the prevention of these episodes by simple adjustment of treatment. In TBI, jugular venous desaturation is mostly related to CBF reduction secondary to decreased CPP (hypotension, intracranial hypertension, and vasospasm) or hypocapnia-associated cerebral vasoconstriction. Studies showed that a sustained reduction of the SjvO2 < 50% was associated with poor outcome, and an independent risk factor for poor prognosis [34-37]. Consequently, SjvO_2 _monitoring is essential for adjustment of ventilation during the medical treatment of an established intracranial hypertension. However, the benefit of SjvO_2 _monitoring on severe TBI patients' outcomes has not been confirmed in a RCT.

#### Brain tissue oxygen tension

Both SjvO_2 _and brain tissue oxygen tension (PbtO_2_) monitoring measure cerebral oxygenation, however, SjvO_2 _measures global cerebral oxygenation and PbtO_2 _measures focal cerebral oxygenation using an invasive probe (Licox). Rosenthal et al. documented that, measurements of PbtO_2 _represent the product of CBF and the cerebral arteriovenous oxygen tension difference rather than a direct measurement of total oxygen delivery or cerebral oxygen [38]. As PbtO_2 _provides a highly focal measurement, it is mainly used to monitor oxygenation of a critically perfused brain tissue. PbtO_2 _is the most reliable technique to monitor focal cerebral oxygenation in order to prevent episodes of desatuartion. However, global cerebral oxygenation alterations may not be observed. The normal PbtO_2 _ranges between 35 mm Hg and 50 mm Hg [39]. A value of a PbtO_2 _< 15 mm Hg is considered a threshold for focal cerebral ischemia and treatment [4]. Several studies demonstrated that PbtO_2_-based therapy may be associated with reduced patient mortality and improved patient outcome after severe TBI [40-42]. In a recent systematic review, available medical literature was reviewed to examine whether PbtO_2_-based therapy is associated with improved patient outcome after severe TBI [43]. Among patients who received PbtO_2_-based therapy, 38.8% had unfavorable and 61.2% had a favorable outcome. Among the patients who received ICP/CPP-based therapy 58.1% had unfavorable and 41.9% had a favorable outcome. Overall PbtO_2_-based therapy was associated with favorable outcome (OR = 2.1; 95% CI = 1.4-3.1). These results suggested that combined ICP/CPP- and PbtO_2_-based therapy is associated with better outcome after severe TBI than ICP/CPP-based therapy alone [43]. Oddo et al. reported that brain hypoxia or reduced PbtO_2 _is an independent outcome predictor and is associated with poor short-term outcome after severe TBI independently of elevated ICP, low CPP, and injury severity. PbtO_2 _may be an important therapeutic target after severe TBI [44]. PbtO_2 _has been documented to be superior to SjvO_2_, near infrared spectroscopy [45], and regional transcranial oxygen saturation [46] in detecting cerebral ischemia. PbtO_2 _monitoring is a promising, safe and clinically applicable method in severe TBI patients; however, it is neither widely used nor available. The combinations of ICP/PbtO2 intra-parenchymal monitoring are important and helpful modalities in the management of severe TBI.

#### Cerebral microdialysis

Cerebral microdialysis (MD) is a recently developed invasive laboratory device, bedside monitor to analyze brain tissue biochemistry [47]. Usually, a MD catheter is inserted in "susceptible" brain tissue to measure biochemical changes in the area of brain most vulnerable to secondary insults. Different assays are available to measure dialysate concentrations including glucose, lactate, pyruvate, glycerol, and glutamate.

Characteristically, cerebral hypoxia or ischemia results in a significant increase in the lactate: pyruvate ratio (LPR) [48]. A LPR > 20-25 is considered a threshold for cerebral ischemia and is associated with poor outcome in TBI [49]. Although, MD is a well-established tool that provides additional assistance in the management of patients with severe TBI, its use is very limited.

#### Transcranial Doppler Ultrasonography

Transcranial Doppler (TCD) is a non-invasive method to measure CBF velocity. It is increasingly utilized in neurocritical care including TBI. It is a clinically useful tool in the diagnosis of complications that may occur in patients with TBI such as vasospasm, critical elevations of ICP and decreases in CPP, carotid dissection, and cerebral circulatory arrest (brain death). TCD can predict post-traumatic vasospasm prior to its clinical manifestations. Since ICP monitoring is an invasive procedure with potential risk of associated complications, TCD has been suggested as a non-invasive alternative technique for assessment of ICP and CPP [50,51]. The overall sensitivity of TCD for confirming brain death is 75% to 88%, and the overall specificity is 98% [52,53]. Although, TCD is an established monitoring modality in neurocritical care, evidence to support its regular use for ICP/CPP management in severe TBI patients is lacking.

#### Electrophysiological monitoring

Electroencephalogram (EEG) is a clinically useful tool for monitoring the depth of coma, detecting non-convulsive (sub-clinical) seizures or seizures activity in pharmacologically paralyzed patients, and diagnosing brain death [54,55]. Continuous EEG has been suggested for the diagnosis of post-traumatic seizures (PTS) in patients with TBI, especially in those who are receiving neuromuscular blockades.

Sensory-evoked potentials (SEP) can yield data on current brain function in very severe TBI patients; however, their use is very limited in the initial management of TBI.

#### Near infrared spectroscopy

Near infrared spectroscopy (NIRS) is a continuous, direct, and non-invasive monitor of cerebral oxygenation and cerebral blood volume (CBV). In cerebral tissue, the two main chromophores (light-absorbing compounds) are hemoglobin (Hb) and cytochrome oxidase. NIRS is based on the differential absorption properties of these chromophores in the NIR range, i.e., between 700 and 1,000 nm. At 760 nm, Hb occurs primarily in the deoxygenated state (deoxyHb), whereas at 850 nm, it occurs in the oxygenated state (oxyHb). Hence, by monitoring the difference in absorbency between these two wavelengths, the degree of tissue deoxygenation can be evaluated.

In comparison with the SjvO_2_, NIRS is less accurate in determining cerebral oxygenation [56]. Although, NIRS is an evolving technology and a potential as a clinical tool for bedside cerebral oxygenation and CBF measurements, its use in neurocritical care remains very limited.

#### Brain temperature

After head trauma, a temperature gradient in brain temperature compared with body temperature of up to 3°C higher in the brain has been reported. Elevated temperature is a common secondary systemic insult to the injured brain. Both invasive (The new Licox PMO: Integra LifeSciences, Plainsboro, NJ) [57] and non-invasive [58], continuous cerebral temperature monitoring devices are commercially available for measuring the brain temperature. However, brain temperature monitoring is still not widely used during neurocritical care of patients with severe TBI.

### Critical Care Management

Guidelines for the management of severe TBI are widely available and should constitute the main background and cornerstone for the development of institutional clinical practice guidelines-based management protocols. Several studies have demonstrated the importance and the impact of implementation of such protocols on the outcomes of patients with severe TBI [5-7]. We reported that the utilization of a clinical practice guidelines-based protocol for severe TBI was associated with a significant reduction in both ICU and hospital mortalities [8].

#### Analgesia, sedation and paralysis

In severe TBI patients, endotracheal intubation, mechanical ventilation, trauma, surgical interventions (if any), nursing care and ICU procedures are potential causes of pain. Narcotics, such as morphine, fentanyl and remifentanil, should be considered first line therapy since they provide analgesia, mild sedation and depression of airway reflexes (cough) which all required in intubated and mechanically ventilated patients. Administration of narcotics is either as continuous infusions or as intermittent boluses.

Adequate sedation potentiates analgesics; provides anxiolysis; limits elevations of ICP related to agitation, discomfort, cough or pain; facilitates nursing care and mechanical ventilation; decrease O_2 _consumption, CMRO_2_, and CO_2 _production; improves patient comfort; and prevents harmful movements. The ideal sedative for TBI patient would be rapid in onset and offset, easily titrated to effect, and lack active metabolites. It would be anticonvulsant, able to lower ICP and CMRO_2_, and to preserve the neurologic examination. Finally, it would lack deleterious cardiovascular effects. No commonly used sedative is ideal. Propofol is the hypnotic of choice in patients with an acute neurologic insult, as it is easily titratable and rapidly reversible once discontinued. These properties permit predictable sedation yet allow for periodic neurologic evaluation of the patient. However, propofol should be avoided in hypotensive or hypovolemic patients because of its deleterious hemodynamic effects. Moreover, propofol infusion syndrome (rhabdomyolysis, metabolic acidosis, renal failure, and bradycardia) is a potential complication of prolonged infusions or high doses of propofol administration. Benzodiazepines such as midazolam and lorazepam are recommended as continuous infusion or intermittent boluses. In addition to sedation, they provide amnesia and anticonvulsive effect. Prolonged infusion, high dose, presence of renal or hepatic failure, and old age are risk factors for accumulation and oversedation.

Routine use of neuromuscular blocking agents (NMBAs) to paralyze patients with TBI is not recommended. NMBAs reduce elevated ICP and should be considered as second line therapy for refractory intracranial hypertension. However, the use of a NMBA is associated with increased risk of pneumonia and ICU length of stay (LOS), and with neuromuscular complications.

#### Mechanical ventilation

Patients with severe TBI are usually intubated and mechanically ventilated. Hypoxia, defined as O_2 _saturation < 90%, or PaO_2 _< 60 mm Hg, should be avoided [4]. Prophylactic hyperventilation to a PaCO_2 _< 25 mm Hg is not recommended [4]. Within the first 24 hours following severe TBI, hyperventilation should be avoided, as it can further compromise an already critically reduced cerebral perfusion. Coles et al. reported that, in patients with TBI, hyperventilation increases the volume of severely hypoperfused tissue within the injured brain, despite improvements in CPP and ICP. These reductions in regional cerebral perfusion may represent regions of potentially ischemic brain tissue [59]. Excessive and prolonged hyperventilation results in cerebral vasoconstriction and ischemia. Thus, hyperventilation is recommended only as a temporizing measure to reduce an elevated ICP. A brief period (15-30 minutes) of hyperventilation, to a PaCO_2 _30-35 mm Hg is recommended to treat acute neurological deterioration reflecting increased ICP. Longer periods of hyperventilation might be required for intracranial hypertension refractory to all treatments including sedation, paralytics, CSF drainage, hypertonic saline solutions (HSSs) and osmotic diuretics. However, when hyperventilation is used, SjvO_2 _or PbtO_2 _measurements are recommended to monitor cerebral oxygenation and avoid cerebral ischemia.

The ventilatory settings should be adjusted to maintain a pulse oximetry (SpO_2_) of 95% or greater and/or PaO_2 _of 80 mm Hg or greater and to achieve normoventilation (eucapnia) with PaCO_2 _of 35 to 40 mm Hg. Mascia et al. reported that high tidal volume ventilation is an independent predictor and associated with acute lung injury (ALI) in patients with severe TBI [60]. Hence, protective ventilation with low tidal volume and moderate positive end-expiratory pressure (PEEP) has been recommended to prevent ventilator-associated lung injury and increased ICP [61].

Prior to suctioning the patient through the endotracheal tube (ETT), preoxygenation with a fraction of inspired oxygen (FiO_2_) = 1.0, and administration of additional sedation are recommended to avoid desaturation and sudden increase in the ICP. Suctioning ETT must be brief and atraumatic.

It has been suggested that PEEP increases intrathoracic pressure leading to a decrease in cerebral venous drainage and consequently to an increase in CBV and ICP. However, the effect of PEEP on ICP is significant only with level of PEEP higher than 15 cm H_2_O in hypovolemic patients. Nevertheless, the lowest level of PEEP, usually 5 to 8 cm H_2_O that maintains adequate oxygenation and prevents end-expiratory collapse, should be used. Higher PEEP, up to 15 cm H_2_O, may be used in cases of refractory hypoxemia.

A significant number of patients with severe TBI develop ALI or acute respiratory distress syndrome (ARDS), with an incidence of ALI/ARDS reported between 10% and 30% [62-64]. Etiology of ALI/ARDS in patients with severe TBI include aspiration, pneumonia, pulmonary contusion, massive blood transfusion, transfusion-related ALI (TRALI), sepsis, neurogenic pulmonary edema and use of high tidal volume and high respiratory rate [65,66]. Development of ALI/ARDS in patients with severe TBI is associated with longer ICU LOS and fewer ventilation free days [60]. Ventilatory management of patients with severe TBI and ALI/ARDS is challenging. A balanced ventilation strategy, between the guidelines for severe TBI or the historical "brain injury" approach (adequate oxygenation: optimizing oxygenation-preserving cerebral venous drainage by using low levels of PEEP, and mild hypocapnia by using high tidal volume), and the lung protective ventilation strategy (by using high PEEP and low tidal volume), is desired, however, is difficult to achieve. Permissive hypercapnia, an acceptable strategy in patients with ALI/ARDS, should be avoided, if possible, in patients with severe TBI because of the associated cerebral vasodilatation, increased CBV and ICP.

#### Hemodynamic support

Hemodynamic instability is common in patients with severe TBI. Hypotension, defined as SBP < 90 mm Hg or MAP < 65 mm Hg, is a frequent and detrimental secondary systemic brain insult and has been reported to occur in up to 73% during ICU stay [67]. Studies from the Traumatic Coma Data Bank (TCDB) documented that hypotension is a major determinant and an independent predictor of outcome of severe TBI (68). Hypotension is significantly associated with increased mortality following TBI [69-71]. Among predictors of outcome of TBI, hypotension is the most amenable to prevention, and should be scrupulously avoided and aggressively managed.

It is unlikely that an isolated TBI by itself would cause hypotension unless the patient has become brain dead. Intravascular volume depletion due to hemorrhage from associated injuries such as scalp, neck, vessels, chest, abdomen, pelvis and extremities, or due to polyuria secondary to diabetes insipidus, are the most common causes of hypotension in patients with severe TBI. Other potential reasons for hypotension in patients with severe TBI are myocardial contusion resulting in primary pump failure, and spinal cord injury with spinal shock (cervical lesions cause total loss of sympathetic innervation and lead to vasovagal hypotension and bradyarrythmias). An often missed cause of hypotension in patients with TBI is the use of etomidate for intubation. It has been reported that even a single dose of etomidate may cause adrenal insufficiency resulting in hypotension [72].

Appropriately aggressive fluid administration to achieve adequate intravascular volume is the first step in resuscitating a patient with hypotension following severe TBI. The CVP may be used to guide fluid management and is recommended to be maintained at 8 - 10 mm Hg. In patients who respond poorly to adequate volume expansion and vasopressors, demonstrate hemodynamic instability, or have underlying cardiovascular disease, a pulmonary artery catheter or non-invasive hemodynamic monitoring may be considered. The pulmonary capillary wedge pressure should be maintained at 12 - 15 mm Hg. Several reliable predictors of fluid responsiveness such as pulse pressure variation, systolic pressure variation, stroke volume variation, and collapse of inferior vena cava have been suggested to guide fluid management. Isotonic crystalloids, specifically normal saline (NS) solution are the fluid of choice for fluid resuscitation and volume replacement. HSSs are effective for blood pressure restoration in hemorrhagic shock; however, with no survival benefit [73]. The National Heart, Lung, and Blood Institute of the National Institutes of Health has stopped enrollment into a clinical trial testing the effects of HSSs on patients with severe TBI because HSS was no better than the standard treatment of NS [74]. Blood and blood products may be used as appropriate.

Anemia is a common secondary systemic brain insult and should be avoided, with a targeted hemoglobin ≥100 g/L or hematocrit ≥0.30. Brain tissue is reach in thromboplastin and cerebral damage may cause coagulopathy [75]. Coagulation abnormalities should be aggressively corrected with blood products as appropriate, especially in the presence of a traumatic intracranial hemorrhage.

Prior to the insertion of an ICP monitoring, a MAP ≥80 mm Hg is recommended. The rationale for a MAP ≥80 mm Hg is to maintain a CPP ≥60 mm Hg for a treatment threshold of ICP > 20 mm Hg [4]. Following the insertion of an ICP monitoring, the management of MAP will be directed by the ICP/CPP values.

Occasionally, targeted CPP or MAP may not be achieved despite appropriate fluid resuscitation and adequate intravascular volume. Excessive and inappropriate fluid administration to achieve intended CPP or MAP is associated with fluid overload and ARDS, and should be avoided. Vasopressors should be used to achieve targeted CPP or MAP if these could not be obtained with adequate fluid resuscitation. Norepinephrine, titrated through a central venous line (CVL), is recommended. Dopamine causes cerebral vasodilatation and increase ICP, however, can be used initially via a peripheral intravenous cannula until a CVL is inserted [76,77]. Phenylephrine, a pure alpha-agonist vasoactive agent, is recommended in TBI patients with tachycardia. A recent study reported that patients who received phenylephrine had higher MAP and CPP than patients who received dopamine and norepinephrine, respectively [78].

Hypertension, defined as SBP > 160 mm Hg or MAP > 110 mm Hg, is also a secondary systemic brain insult that can aggravate vasogenic brain edema and intracranial hypertension. However, hypertension may be a physiological response to a reduced cerebral perfusion. Consequently, and prior to ICP monitoring, hypertension should not be treated unless a cause has been excluded or treated, and SBP > 180-200 mm Hg or MAP > 110-120 mm Hg. Lowering an increased BP, as a compensatory mechanism to maintain an adequate CPP, exacerbates cerebral ischemia. Following placement of an ICP monitoring, the management of MAP is guided by the CPP.

#### Cerebral perfusion pressure

Cerebral ischemia is considered the single most important secondary event affecting outcome following severe TBI. CPP, defined as the MAP minus ICP, (CPP = MAP - ICP), below 50 mm Hg should be avoided [4]. A low CPP may jeopardize regions of the brain with pre-existing ischemia, and enhancement of CPP may help to avoid cerebral ischemia. The CPP value to target should be maintained above the ischemic threshold at a minimum of 60 mm Hg [4]. Maintenance of a CPP greater than 60 mmHg is a therapeutic option that may be associated with a substantial reduction in mortality and improvement in quality of survival, and is likely to enhance perfusion to ischemic regions of the brain following severe TBI. There is no evidence that the incidence of intracranial hypertension, morbidity, or mortality is increased by the active maintenance of CPP above 60 mmHg with normalizing the intravascular volume or inducing systemic hypertension. Both 60 mm Hg and 70 mm Hg are cited in the literature as the threshold above which CPP should be maintained. The CPP should be maintained at a minimum of 60 mm Hg in the absence of cerebral ischemia, and at a minimum of 70 mm Hg in the presence of cerebral ischemia [4]. PbtO_2 _monitoring has been suggested to identify individual optimal CPP [79]. In the absence of cerebral ischemia, aggressive attempts to maintain CPP above 70 mm Hg with fluids and vasopressors should be avoided because of the risk of ARDS [4].

#### Hyperosmolar therapy

Mannitol administration is an effective method to decrease raised ICP after severe TBI [80]. Mannitol creates a temporary osmotic gradient and it increases the serum osmolarity to 310 to 320 mOsm/kg H_2_O. The prophylactic administration of mannitol is not recommended [4]. Prior to ICP monitoring, mannitol use should be restricted to patients with signs of transtentorial herniation or progressive neurologic deterioration not attributable to extracranial causes. Arbitrarily, mannitol should not be administered if serum osmolarity is > 320 mOsm/kg H_2_O. Osmotic diuresis should be compensated by adequate fluid replacement with isotonic saline solution to maintain euvolmia. The effective dose is 0.25-1 g/kg, administered intravenously over a period of 15 to 20 minutes. The regular administration of mannitol may lead to intravascular dehydration, hypotension, pre-renal azotemia and hyperkalemia [81]. Mannitol may pass and accumulate in the brain, causing a reverse osmotic shift or rebound effect, and raising brain osmolarity, thus increasing ICP [82,83]. Mannitol is contraindicated in patients with TBI and renal failure because of the risk of pulmonary edema and heart failure.

HSSs have been suggested as alternative to mannitol. HSS has a number of beneficial effects in head-injured patients, including expansion of intravascular volume, extraction of water from the intracellular space, decrease in ICP, and increase in cardiac contractility. HSS produces osmotic dehydration and viscosity-related cerebral vasoconstriction. Prolonged administration of a HSS was associated with lowered ICP, controlled cerebral edema, with no adverse effects of supraphysiologic hyperosmolarity such as renal failure, pulmonary edema, or central pontine demyelination [84,85]. In a recent meta-analysis, Kamel et al. found that hypertonic saline is more effective than, and may be superior to the current standard of care which is, mannitol for the treatment of elevated ICP [86].

#### Temperature Modulation

Moderate systemic hypothermia at 32°C to 34°C, reduces cerebral metabolism and CBV, decreases ICP, and increases CPP [87]. Evidence of the impact of moderate hypothermia on the outcome of patients with TBI was controversial. Initially, studies showed that moderate hypothermia, established on admission, was associated with significantly improved outcome at 3 and 6 months after TBI [88]. However, in a large RCT, no effect of moderate hypothermia has been demonstrated on outcome after TBI [89,90]. The National Acute Brain Injury Study: Hypothermia II was a randomized, multicentre clinical trial of patients with severe TBI who were enrolled within 2 to 5 hours of injury. Patients were randomly assigned to hypothermia (cooling to 33°C for 48 hours) or normothermia. There was no significant difference in outcomes between the hypothermia and the normothermia groups. The trial did not confirm the utility of hypothermia as a primary neuroprotective strategy in severe TBI patients [88]. However, temperature should be controlled and fever should be aggressively treated in patients with severe TBI. Moderate hypothermia may be used in refractory, uncontrolled ICP.

#### Antiseizure prophylaxis

Post-traumatic seizures are classified as early occurring within 7 days of injury, or late occurring after 7 days following injury [91]. Prophylactic therapy (phenytoin, carbamazepine, or phenobarbital) is not recommended for preventing late post-traumatic seizures [4]. However, the BTF recommended prophylaxis therapy to prevent early post-traumatic seizure in TBI patients who are at high risk for seizures [4]. The risk factors include: GCS score < 10, cortical contusion, depressed skull fracture, subdural hematoma, epidural hematoma, intracerebral hematoma, penetrating TBI, and seizures within 24 hours of injury [4,92].

Phenytoin is the recommended drug for the prophylaxis of early post-traumatic seizures. A loading dose of 15 to 20 mg/kg administered intravenously (I.V.) over 30 minutes followed by 100 mg, I.V., every 8 hours, titrated to plasma level, for 7 days, is recommended. Patients receiving antiseizures prophylaxis should be monitored for potential side effects.

#### Deep vein thrombosis prophylaxis

Severe TBI patients are at significantly high risk of developing venous thromoembolic events (VTEs) including deep vein thrombosis (DVT) and pulmonary embolism. The risk of developing DVT in the absence of prophylaxis was estimated to be 20% after severe TBI [93].

Mechanical thromboprophylaxis, including graduated compression stockings and sequential compression devices, are recommended unless their use is prevented by lower extremity injuries. The use of such devices should be continued until patients are ambulatory. In the absence of a contraindication, low molecular weight heparin (LMWH) or low dose unfractionated heparin should be used in combination with mechanical prophylaxis. However, the use of pharmacological prophylaxis is associated with an increased risk for expansion of intracranial hemorrhage. Although, evidence to support recommendations regarding the timing of pharmacological prophylaxis is lacking, most experts suggest initiating pharmacologic prophylaxis as early as 48 to 72 hours after the injury, in the absence of other contraindications [94].

#### Stress ulcer prophylaxis

Severe TBI is a well-known risk factor for stress ulcers (Cushing's ulcer) in the ICU. Prophylaxis includes early enteral feeding, and pharmacological prophylaxis such as H2- blockers, proton-pump inhibitors and sucralfate [95,96].

#### Nutritional support

Severe TBI patients are usually in hypermetabolic, hypercatabolic and hyperglycemic state, with altered G.I. functions. There is evidence suggesting that malnutrition increases mortality rate in TBI patients [97]. Studies documented the superiority of enteral feeding over parenteral nutrition (PN). Use of PN should be limited to contraindications of enteral feeding, as it is associated with complications and an increased mortality [98]. Hence, early enteral feeding is recommended in patients with severe TBI, as it is safe, cheap, cost-effective, and physiologic. The potential advantages of enteral feeding include stimulation of all gastro-intestinal tract functions, preservation of the immunological gut barrier function and intestinal mucosal integrity, and reduction of infections and septic complications. Frequently, patients with severe TBI have gastric feeding intolerance due to many reasons including abnormal gastric emptying and altered gastric function secondary to increased ICP, and use of opiates. Prokinetic agents such as metoclopramide or erythromycin, improve tolerance. Post-pyloric feeding avoids gastric intolerance and allows higher caloric and nitrogen intake.

Although, the BTF recommended 140% of resting metabolic expenditure in non-paralyzed patients and 100% in paralyzed patients to be replaced, there is growing body of evidence suggesting the benefit of a lower caloric intake [99-102].

#### Glycemic control

In patients with severe TBI, stress hyperglycemia is a common secondary systemic brain insult. Studies showed that hyperglycemia has repeatedly been associated with poor neurological outcome after TBI [103-108]. Although hyperglycaemia is detrimental, maintaining low blood glucose levels within tight limits is controversial in patients with severe TBI, because hypoglycemia, a common complication of tight glucose control, can induce and aggravate underlying brain injury [109]. Vespa et al. reported that intensive insulin therapy (IIT) results in a net reduction in microdialysis glucose and an increase in microdialysis glutamate and lactate/pyruvate ratio without conveying a functional outcome advantage [110]. Oddo et al. documented that tight systemic glucose control is associated with reduced cerebral extracellular glucose availability and increased prevalence of brain energy crisis, which in turn correlates with increased mortality. IIT may impair cerebral glucose metabolism after severe brain injury [111]. A recent meta-analysis on IIT in brain injury revealed that IIT did not appear to decrease the risk of in-hospital or late mortality (RR = 1.04, 95% CI = 0.75, 1.43 and RR = 1.07, 95%CI = 0.91, 1.27 respectively). Moreover, IIT did not have a protective effect on long-term neurological outcomes (RR = 1.10, 95% CI = 0.96, 1.27). However, IIT increased the rate of hypoglycemic episodes (RR = 1.72, 95% CI = 1.20, 2.46) [112]. Consequently, the majority of currently available clinical evidence does not support tight glucose control (maintenance of blood glucose levels below 110-120 mg/dl) during the acute care of patients with severe TBI [113].

#### Steroids

Steroids administration is not recommended for improving the outcome or reducing ICP in patients with severe TBI. Moreover, steroids may be harmful after TBI. The CRASH trial, a multicentre international collaboration, aimed to confirm or refute such an effect by recruiting 20000 patients. In May, 2004, the data monitoring committee disclosed the unmasked results to the steering committee, which stopped recruitment at 10008 patients. Compared with placebo, the risk of death from all causes within 2 weeks was higher in the group allocated corticosteroids (1052 [21.1%] vs. 893 [17.9%] deaths; relative risk = 1.18 [95% CI = 1.09-1.27]; p = 0.0001). The authors concluded that there was no reduction in mortality with methylprednisolone in the 2 weeks after head injury. The cause of the rise in risk of death within 2 weeks was unclear [114]. Hence, in patients with severe TBI, high-dose methylprednisolone is contraindicated [4].

#### Barbiturate coma

Barbiturate is proven as efficient therapy for refractory intracranial hypertension. Barbiturates reduce cerebral metabolism and CBF, and lower ICP [115]. High-dose barbiturate may be considered in hemodynamically stable, severe TBI patients with refractory to maximal medical and surgical ICP lowering therapy. Their main side effects are: hypotension, especially in volume depleted patients; and immunosuppression with an increased infection rate [116]. However, prophylactic administration of barbiturate to induce burst suppression EEG is not recommended [4]. Pentobarbital is recommended for the induction of barbiturate coma as follows:

Pentobarbital: 10 mg/kg over 30 min, then

5 mg/kg/h for 3 hours, then

1 mg/kg/h

As alternative, sodium thiopental might be used as follows:

2.5-10 mg/kg IV, slow bolus, then

0.5-2 mg/kg/h

#### Fluids and electrolytes

The goal of fluid management is to establish and maintain euvolemia to moderate hypervolemia (CVP = 8 - 10 mm Hg; PCWP = 12 - 15 mm Hg). Negative fluid balance has been shown to be associated with an adverse effect on outcome, independent of its relationship to ICP, MAP, or CPP [117]. Isotonic crystalloids should be used for fluid management, and normal saline (NS) is the recommended solution. Aggressive fluid resuscitation with NS may result in hyperchloremic metabolic acidosis, a predictable and important consequence of large-volume, saline-based intravenous fluid administration, with different clinical implications. Hypotonic solutions, such as 1/2 NS, ¼ NS, Dextrose 5% in water (D5%W), D5% 1/2 NS, or D5% ¼ NS should be avoided. Ringer's lactate solution is slightly hypotonic and is not preferred for fluid resuscitation in severe TBI patients, particularly for large volume resuscitation, as it may decrease serum osmolarity. Glucose containing solutions, as above or D10%W should be avoided in the first 24 to 48 hours, unless the patient develops hypoglycemia in the absence of nutritional support. In addition to the detrimental effects of hyperglycemia in TBI, anaerobic cerebral metabolism of glucose produces acidosis and free water; both would worsen the brain edema. The use of colloids should be very cautious as it was reported, in the SAFE trial, to be associated with increased mortality in patients with TBI [118]. HSSs have been shown to be effective in decreasing brain edema, reducing elevated ICP, and increasing MAP and CPP [119]. Other potential benefits of HSSs include faster expansion of intravascular volume (with small volumes), increased cardiac output and pulmonary gas exchange, reversal of immunomodulation caused by hypotension, and decreased CSF production. HSS is also associated with potential side effects including sudden hypertension, hypernatremia, altered consciousness and seizures. However, the overall results of HSS related studies are inconsistent and further clinical trials are needed to define its role.

In severe TBI patients with increased ICP or brain edema, a serum sodium level Na^+ ^up to 150 - 155 mEq/L may be acceptable [120]. However, serum electrolytes disturbances are common complications after TBI. Injury to the hypothalamic-pituitary system is a major contributing factor. The most common causes for hypernatremia (Na^+ ^> 150 mmol/L) in patients with TBI are central or neurogenic diabetes insipidus, osmotic diuresis (mannitol), and the use of HSS. Correction of severe hypernatremia (Na^+ ^> 160 mmol/L) should be gradual, as abrupt changes in serum osmolarity and rapid fall of serum sodium concentration would worsen cerebral edema. Fluid resuscitation of hypovolemic hypernatremic TBI patients should be initially only with NS. Management of electrolytes disturbances should follow complete volume restoration. Hyponatremia is detrimental and major secondary systemic brain insult in patients with severe TBI, as it leads to exacerbation of brain edema and an increase in ICP. It is usually secondary to cerebral salt wasting syndrome [121], or to the syndrome of inappropriate anti-diuretic hormone secretion (SIADH). Hypophosphatemia and hypomagnesemia are common complications in head-injured patients and they lower the seizure threshold [122,123].

#### Lund therapy

The "Lund therapy" of severe TBI is based on physiological principles for cerebral tissue and blood volume regulation. The therapy aims at preventing cerebral hypoxia simultaneously with taking measures that counteract transcapillary filtration. The Lund concept is more beneficial if the blood brain barrier is disrupted and more appropriate if pressure autoregulation is lost. The therapy has two main goals: first to reduce or prevent an increase in ICP (ICP-targeted goal), and second to improve perfusion and oxygenation around contusions (perfusion-targeted goal) by maintaining normal blood oxygenation, normovolemia and normal hematocrit. The treatment protocol, to reduce an increased ICP, includes preservation of a normal colloidal absorbing force (normal plasma protein concentrations), a reduction of intracapillary pressure through reduction of systemic blood pressure by antihypertensive therapy (a beta1-antagonist, metoprolol, combined with an alpha 2-agonist, clonidine) and a simultaneous, moderate constriction of precapillary resistance vessels with low-dose thiopental and dihydroergotamine. A few studies have reported that Lund therapy was associated with improved clinical outcome [124]

#### General intensive care

Similar to other patients in the intensive care, TBI victims should receive the usual daily care as follows:

- Raising head of bed to 30° - 45°: that would reduce ICP and improves CPP [125]; and lower the risk of ventilator-associated pneumonia (VAP).

- Keeping the head and neck of the patient in a neutral position: this would improve cerebral venous drainage and reduce ICP.

- Avoiding compression of internal or external jugular veins with tight cervical collar or tight tape fixation of the endotracheal tube that would impede cerebral venous drainage and result in an increase in the ICP.

- Turning the patient regularly and frequently with careful observation of the ICP [126].

- Providing eye care, mouth and skin hygiene

- Implementing all evidence-based bundles for prevention of infection including VAP [127] and central line bundle [128].

- Administrating a bowel regimen to avoid constipation and increase of intra-abdominal pressure and ICP.

- Performing physiotherapy

#### Decompressive craniectomy and hemicraniectomy

Surgical decompressive craniectomy has been suggested as a promising therapeutic approach for patients with acute severe TBI at risk to develop severe brain edema. Decompressive craniectomy and hemicraniectomy, both are well accepted for the surgical treatment of intractable intracranial hypertension in cases in which medical management fails. Decompressive surgery is performed as a life-saving procedure when death is imminent from intracranial hypertension. Though the operation is being increasingly used, evidence regarding its overall effects on outcomes is contradicting. Albanèse et al, in a retrospective cohort study in 40 patients with intractable intracranial hypertension and at very high risk of brain death, decompressive craniectomy allowed 25% of patients to attain social rehabilitation at 1 yr [129]. Cooper et al, in a prospective, randomized controlled trial in 155 adults with severe diffuse TBI and intracranial hypertension that was refractory to first-tier therapies, bifrontotemporoparietal decompressive craniectomy, as compared with standard care, was associated with decreased intracranial pressure (P < 0.001) and length of stay in the ICU (P < 0.001), however, with more unfavorable outcomes (odds ratio = 2.21; 95% CI = 1.14 - 4.26; P = 0.02). Rates of death at 6 months were similar in the craniectomy group (19%) and the standard-care group (18%) [130].

### Predicting outcome after TBI

The early prediction of outcome after TBI is important. A few predictive models for patient outcomes after severe TBI have been proposed [131,132]. A relatively simple prognostic model using 7 predictive baseline characteristics including age, motor score, pupillary reactivity, hypoxia, hypotension, computed tomography classification, and traumatic subarachnoid hemorrhage has been reported to accurately predict 6-month outcome in patients with severe or moderate TBI [131]. A predictive model based on age, absence of light reflex, presence of extensive subarachnoid hemorrhage, ICP, and midline shift was shown to have high predictive value and to be useful for decision making, review of treatment, and family counseling in case of TBI [132].

## Conclusion

The management of severe TBI centers on meticulous and comprehensive intensive care that includes multi-model, protocolized approach involving careful hemodynamic support, respiratory care, fluid management, and other aspects of therapy, aimed at preventing secondary brain insults, maintaining an adequate CPP, and optimizing cerebral oxygenation. This approach clearly requires the efforts of a multidisciplinary team including neurointensivists, neurosurgeons, bedside nurses and respiratory therapists, and other members of the medical team. While such management can be challenging, it is by all means rewarding considering the age of the victims and the socio-economic impact of the problem.

## List of abbreviations

BTF: Brain Trauma Foundation; CBF: Cerebral blood flow; CBV: Cerebral blood volume; CPP: Cerebral perfusion pressure; CSF: Cerebral spinal fluid; CVP: Central venous pressure; EEG: Electroencephalogram; GCS: Glasgow coma scale; HSS: Hypertonic saline solution; ICP: Intracranial pressure; MAP: Mean arterial pressure; NS: Normal saline; PbtO_2_: Brain tissue oxygen tension; PEEP: Positive end expiratory pressure; SBP: Systolic blood pressure; SIADH: Syndrome of inappropriate anti-diuretic hormone secretion; SjvO_2_: Jugular venous oxygen saturation; TBI: Traumatic brain injury.

## Competing interests

The authors declare that they have no competing interests.

## Authors' contributions

SHH performed literature review and wrote the initial draft of the manuscript. YMA edited and rewrote portions of the manuscript. All authors read and approved the final manuscript.

## Authors' information

Samir H. Haddad, MD, is Head Section of Surgical Intensive Care Unit; and Consultant in the Intensive Care Department at King Abdulaziz Medical City, Riyadh, Saudi Arabia.

Yaseen M. Arabi, MD, FCCP, FCCM, is Chairman, Intensive Care Department; and Medical Director, Respiratory Services at King Abdulaziz Medical City, Riyadh, Saudi Arabia. He is also Associate Professor at College of Medicine, King Saud Bin Abdulaziz University for Health Sciences, Riyadh, Saudi Arabia.
